# Hippocampal expression of cell‐adhesion glycoprotein neuroplastin is altered in Alzheimer's disease

**DOI:** 10.1111/jcmm.13998

**Published:** 2018-11-28

**Authors:** Katarina Ilic, Kristina Mlinac‐Jerkovic, Natasa Jovanov‐Milosevic, Goran Simic, Nikola Habek, Nenad Bogdanovic, Svjetlana Kalanj‐Bognar

**Affiliations:** ^1^ School of Medicine Croatian Institute for Brain Research University of Zagreb Zagreb Croatia; ^2^ Division of Clinical Geriatrics Department of Neurobiology, Care Sciences and Society Karolinska Institutet Stockholm Sweden

**Keywords:** cell‐adhesion molecules, human hippocampus, immunohistochemistry, neurodegeneration

## Abstract

Cell‐adhesion glycoprotein neuroplastin (Np) is involved in the regulation of synaptic plasticity and balancing hippocampal excitatory/inhibitory inputs which aids in the process of associative memory formation and learning. Our recent findings show that neuroplastin expression in the adult human hippocampus is specifically associated with major hippocampal excitatory pathways and is related to neuronal calcium regulation. Here, we investigated the hippocampal expression of brain‐specific neuroplastin isoform (Np65), its relationship with amyloid and tau pathology in Alzheimer's disease (AD), and potential involvement of neuroplastin in tissue response during the disease progression. Np65 expression and localization was analysed in six human hippocampi with confirmed AD neuropathology, and six age‐/gender‐matched control hippocampi by imunohistochemistry. In AD cases with shorter disease duration, the Np65 immunoreactivity was significantly increased in the dentate gyrus (DG), *Cornu Ammonis 2/3* (CA2/3), and subiculum, with the highest level of Np expression being located on the dendrites of granule cells and subicular pyramidal neurons. Changes in the expression of neuroplastin in AD hippocampal areas seem to be related to the progression of disease. Our study suggests that cell‐adhesion protein neuroplastin is involved in tissue reorganization and is a potential molecular marker of plasticity response in the early neurodegeneration process of AD.

## 1 INTRODUCTION

Studies in literature have indicated multiple different (patho)physiological functions of cell‐adhesion molecule (CAM) neuroplastin in the human brain such as: the association of neuroplastin gene polymorphisms with cognitive abilities and cortical thickness in adolescents^1^; single nucleotide polymorphisms that are associated with a higher risk of developing schizophrenia.^2^ Also, we recently reported that immunohistochemical localization of neuroplastin in the adult human hippocampus specifically delineates hippocampal circuitry and its principal excitatory pathways, and that there is a link between Np expression and calcium regulation in murine cortical hippocampal glutamatergic neurons.^3^ Two isoforms of neuroplastin, Np55 and brain‐specific Np65, have been described.^4^ Neuroplastin functions in several vital processes in the mammalian central nervous system including neurite outgrowth,^5^ regulation of synaptic plasticity,^6^ long‐term potentiation,^7^, ^8^ maintaining balance between the excitatory and inhibitory pathways^9^ and the formation of associative memory.^10^ Data from mice and rats have allowed us to determine that neuroplastin has a high preference for the hippocampus and cerebellum,^11^, ^12^ but only two studies have systematically analysed Np expression in human brains.^3^, ^11^ As with other CAMs, it is presumed that Np is involved in the molecular events which underlay the structural and functional processes of brain development, ageing, and neurodegeneration.^13^, ^14^, ^15^ The adult human hippocampus retains a neuroplastic potential which enables it to remodel and reorganize after injury.^16^ This has led us to believe that neuroplastin expression changes during neurodegeneration as well. Therefore, in this study we analysed the expression and distribution of neuroplastin immunoreactivity in human hippocampal sections derived from brains of individuals with Alzheimer's disease (AD) and control sections obtained from cognitively normal subjects. We found increased neuroplastin immunoreactivity in all major hippocampal areas (Ammon's horn, dentate gyrus, subiculum) affected by AD pathology when compared to age‐/gender‐matched controls. This strongly indicates that CAM neuroplastin is involved in molecular events underlying tissue response in neurodegeneration.

## 2 MATERIALS AND METHODS

### 2.1 Human brain samples

Immunohistochemical analyses were performed on hippocampal sections derived postmortem from: (a) individuals with neuropathologically confirmed AD (N = 6; mean age 77.8, age range: 73‐84 years; clinically assessed mean duration of AD: 4.5 years, disease duration range: 3.5‐7 years); (b) age‐/gender‐matched control subjects with a negative history of neuropsychiatric disorders and cognitive deficits, and cause of death not related to a neurological disorder or head trauma (N = 6; mean age: 75.5, age range: 62‐84 years). All brain sections were obtained from the Huddinge Brain Bank (Karolinska Institutet, Stockholm, Sweden), in accordance with ethical requirements and legislative permissions (Table S1).

### 2.2 Neuroanatomical, histochemical, immunohistochemical, and volumetric procedures

All brains were fixed in 10% neutral formalin for 21 days prior to paraffin embedding. Left hippocampi were cut in rostrocaudal direction, with a random position for the first cut within the first rostral 3 mm, as previously described.^17^ Twelve micrometers thick sections were used for both Nissl staining and immunohistochemistry. The following procedures were performed, as previously published: (a) assessment of the tissue shrinkage; (b) estimation of the number of hippocampal neurons; (c) delineation and volumetric estimation of hippocampal subfields; (d) Braak staging and neurofibrillary tangles (NFT) counting.^18^, ^19^

For immunohistochemistry, sections were dewaxed and rehydrated. Following the antigen retrieval in citrate buffer (pH 6.0 at 95°C for 30 minutes) and pretreatment with 2.25% hydrogen peroxide in methanol and water for 30 minutes, sections were incubated in blocking solution (5% horse serum and 0.5% Triton X‐100 in PBS) for 2 hours at RT. Incubation with primary anti‐neuroplastin 65 antibody raised in goat (1:100, R&D Systems, AF5360, Minneapolis, Minnesota, USA) in blocking solution was performed at +4°C overnight. Parallel sections incubated in blocking solution without primary antibody were used as negative controls. Incubation in secondary anti‐goat antibody conjugated with horse‐radish peroxidase (Jackson ImmunoResearch Laboratories, West Grove, Pennsylvania, USA) in blocking solution was performed at RT for 2 hours. Diaminobenzidine (DAB) was used as an enhancement agent for immunoreactivity visualization. Sections were scanned using a high‐resolution scanner (Hamamatsu NanoZoomer C10730‐12). Signal intensities were semiquantitatively scored by three independent researchers as none (0), low (+), moderate (++), or strong (+++) (Table S2). Final confirmation and quantification of Np immunoreactivity signal was done using *ImageJ* densitometric analysis (*ImageJ*, NIH public domain, https://imagej.nih.gov/ij/). For the image calibration, a calibrated optical density step tablet was used according to instructions. The intensity of the immunoreaction on each slide was additionally corrected for alveus immunoreactivity, the white matter area showing no reaction for neuroplastin.

The volume and number of neurons were assessed stereologically, as described previously.^19^ Briefly, following manual delineation of the hippocampal formation areas at low magnification, estimates of the reference volume for each area were made using the Cavalieri principle. For determination of the shrinkage in the third dimension (assuming that shrinkage is similar in all three dimensions), we used the value of the square root of the areal shrinkage as a correction factor on slab thickness used in the Cavalieri formula.

Next, the numerical density of neurons was measured using the disector method.^20^ Finally, the total number of neurons was obtained by multiplying the numerical density of the particular hippocampal subdivision with its reference volume.

### 2.3 Statistical analysis

Statistical analysis of total Np immunoreactivity was done by using the Student's *t* test. Np immunoreaction intensities in different AD hipocampal areas were grouped according to disease duration (less than four and more than 5 years), and were compared to controls by One‐Way ANOVA and Tukey post‐hoc analysis. Relationship between the number of amyloid plaques and neurofibrillary tangles in major hippocampal areas with quantified Np total immunoreactivity was analysed using Pearson's correlation coefficient. All statistics were performed by using IBM SPSS ver. 25 (IBM Analytics, New York, NY, USA) software.

## 3 RESULTS

### 3.1 Hippocampal expression of neuroplastin in Alzheimer's disease

We found altered levels of neuroplastin expression in distinct sublayers of the human hippocampus in AD brains. When compared to age‐matched control tissues, we noticed that the overall intensity of neuroplastin immunostaining was higher in the AD hippocampi of early stage diseased patients and that the distribution of Np in sublayers of the hippocampi had changed.

**Figure 1:**
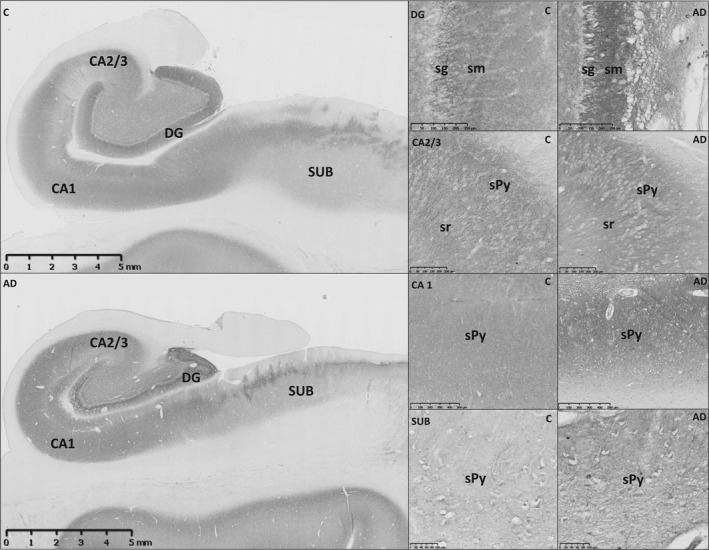
Neuroplastin immunoreactivity pattern in hippocampal sections derived from individuals with neuropathologically confirmed Alzheimer's disease (AD) and age/gender‐matched controls. Representative data are shown, observed on at least three sections derived from six AD and six control hippocampi. Immunoreactivity of neuroplastin is increased in all major analysed hippocampal areas (dentate gyrus, CA1, CA2/CA3, and subiculum) of AD sections. Different intensity of specific neuroplastin immunostaining was demonstrated at the cellular level between control and AD sections, as well as the association of neuroplastin immunostaining with bodies and dendrites of granule cells in the inner molecular layer of dentate gyrus, pyramidal neurons of CA2/3, CA1 and subiculum. C, control; AD, Alzheimer's disease; DG, dentate gyrus; CA1, CA2/CA3, *Cornu Ammonis*; SUB, subiculum; sg, stratum granulare; sm, stratum moleculare; sPy, stratum pyramidale. Scale bars ‐ 5 mm (hippocampal formation); 100 μm (subiculum), 250 μm (DG and CA2/3), 500 μm (CA1)

Human Np65 was found to specifically localize on the neuronal membranes and in neuropil, while the overall Np immunoreactivity is distributed throughout distinct neuron‐containing hippocampal sublayers in both AD and control hippocampi (Figure 1). The highest Np signal in normal human hippocampus is present in the CA1 area and dentate gyrus, > CA2/3, > hilus, > subiculum, and is specifically expressed in sublayers containing pyramidal or granule cells, as reported previously.^3^

In AD hippocampi, a convincing increase in Np immunoreactivity intensity has been consistently found in all major analysed hippocampal areas (Figure 1). At the cellular level, the change in Np immunoreactivity in AD hippocampi was found to be associated mostly with neurons of the pyramidal sublayer in CA1, CA2/3, and subiculum, and with DG granule cells (Figure 1; Table S2). In the dentate gyrus, hippocampal area known to be a site of adult neurogenesis and intensive plasticity,^16^ increased Np immunoreactivity was predominantly associated with dendrites of granule cells in the inner molecular layer (Figure 1). Additional detected changes of Np expression in AD hippocampi included accumulation of immunoreactive intracellular structures in the subicular pyramidal layer (Figure 1).

**Figure 2:**
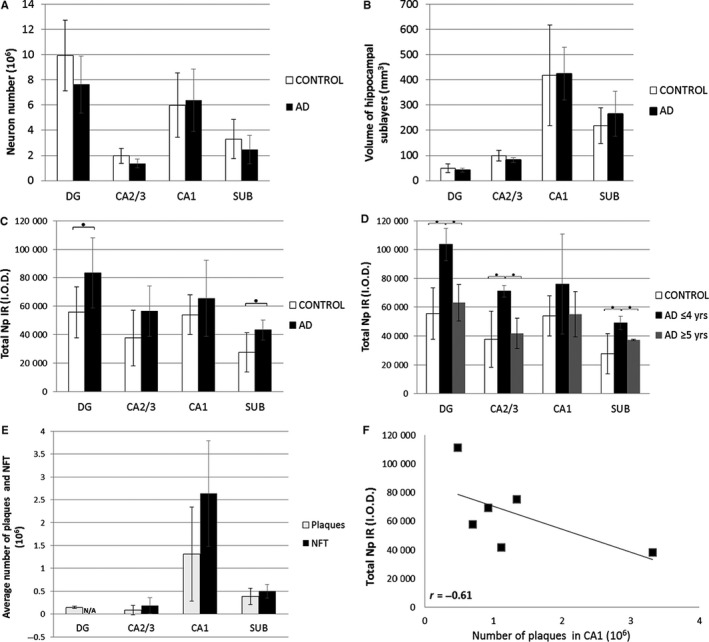
Neuroplastin immunoreactivity in human hippocampus changes both with ageing in controls and progression of Alzheimer's disease. No significant quantitative difference in (A) neuron number and (B) volumes of hippocampal areas in AD sections was found when compared with controls. (Error bars denote SD, standard deviation around mean of data referring to volumetric parameters.) (C) Quantification of neuroplastin immunoreactivity in analysed hippocampal areas, expressed as total neuroplastin immunoreactivity revealed increased values in AD compared with controls, being statistically significant in dentate gyrus and subiculum. Error bars denote standard deviation (SD) around mean of data referring to quantified Np immunoreactivity in sections derived from six AD and six control subjects. Asterisk (*), *P* < 0.05, Student *t* test. (D) In AD, total neuroplastin immunoreactivity tends to decrease with disease progression in all analysed hippocampal areas, however, still remains higher in AD than in control hippocampal sections. Significant difference in Np immunoreactivity was found when comparing controls and AD with shorter disease duration in DG, CA2/3 and subiculum, as well as when comparing two AD groups differing in disease duration (asterisk denotes *P* < 0.05, One Way ANOVA and Tukey post‐hoc analysis; error bars denote standard deviation (SD). (E) Quantification of amyloid plaques and neurofibrillary tangles in AD hippocampal sections confirmed the CA1 as the area with the highest burden of specific AD neuropathological changes. (F) In AD hippocampal sections, negative correlation was found for Np immunoreactivity and number of amyloid plaques in CA1 (*r *= –0.61, Pearson's). Np, neuroplastin; IR, immunoreactivity; I.O.D., integrated optical density; DG, dentate gyrus; CA1, CA2/3, Cornu Ammonis; SUB, subiculum; AD, Alzheimer's disease; NFT, neurofibrillary tangles; N/A, not assessed

In order to investigate whether cytopathological changes in AD may interfere with our interpretation of Np immunoreactivity, we performed quantitative analysis of neuron number and measurement of volume of hippocampal areas in AD and matched control sections. Neither of these measured parameters was shown to be significantly different between AD and controls, supporting our finding of increased total hippocampal Np immunoreactivity in AD vs controls by excluding a potential influence of factors such as changed cell number or tissue volume on the interpretation of Np immunoreactivity (Figure 2A, B). Indeed, quantification of the overall Np signal intensities revealed: (a) significantly increased total Np immunoreaction in AD hippocampi compared to controls in dentate gyrus and subiculum (*P* = 0.049 and 0.034, respectively) (Figure 2C); (2) significantly increased Np immunoreactivity expressed per neuron number in dentate gyrus and CA2/3 (*P* = 0.018, 0.031, respectively).

### 3.2 Neuroplastin expression in the hippocampus changes with progression of Alzheimer's disease

We observed another interesting feature of hippocampal Np expression related to the changes in Np immunoreactivity intensity with ageing in controls and with disease progression in AD. First, quantification of total Np immunoreactivity confirmed higher Np signal intensities in all AD samples than in controls (Figure 2C). Second, the hippocampal Np signal intensity was significantly higher in AD with shorter disease duration (≤4 years) vs age‐matched controls in dentate gyrus, CA2/3, and subiculum (*P* = 0.004, 0.025, 0.038, respectively) (Figure 2D). Third, although total Np immunoreactivity had been persistently higher in AD hippocampus and there was a statistical difference found in specific hippocampal areas of early stage disease vs controls, our results indicate that total Np expression decreases with ageing in controls (data not shown) and in the AD group with longer disease duration (5‐7 years) (Figure 2D). Moreover, the comparison of quantified neuroplastin immunoreactivity between two AD groups with a different disease duration (AD ≤ 4 years vs AD ≥5 years) revealed a significant decrease in Np immunoreactivity with disease progression in the dentate gyrus, CA2/3, and subiculum, (*P* = 0.014, 0.010, 0.011, respectively) (Figure 2D).

We further investigated the relationship of neuropathological features specific for neurodegeneration with detected alterations of neuroplastin immunoreactivity in AD hippocampi. The highest number of amyloid plaques and neurofibrillary tangles were found in CA1 (Figure 2E). A strong negative correlation was found between neuroplastin immunoreactivity and the number of amyloid plaques in CA1 (*r *=* *–0.61) (Figure 2F). Weak negative correlation was observed between the NFT number and neuroplastin immunoreactivity in CA1 (*r *=* *–0.33), CA2/3 (*r *=* *–0.15) and subiculum (*r *=* *–0.17).

## 4 DISCUSSION

Using immunohistochemical techniques, this study showed that the expression of neuroplastin, CAM known to be involved in processes of learning, memory and cognition, is consistently and significantly changed in the major hippocampal areas in Alzheimer's disease. Observed alterations of hippocampal Np immunoreactivity are clearly related to both the distribution and dynamics of pathological events in AD. Our study suggests that the aberrations of Np expression in these hippocampal areas are because of a response to attenuate the neuropathological changes caused by AD. This finding supports previously reported literature entailing alterations of CAMs in response to lesions that trigger a neurodegenerative pathological cascade.^14^, ^15^, ^21^, ^22^

Significantly increased Np expression in early‐phase AD may reflect structural and functional reorganization of the tissue, while reduction in hippocampal compensatory cellular and molecular capacities during disease progression is associated with an overall decreased Np immunostaining intensity. Our study demonstrated that in the early stage of AD the total Np expression increases predominantly in the dentate gyrus, mainly unaffected by NFT.^23^ As the disease progresses, Np immunoreactivity is still higher in the DG than in age‐matched controls, however, the overall signal intensity decreases in all analysed areas and most dramatically in CA1, a vulnerable hippocampal area with the highest amount of amyloid plaques and NFT burden.^23^ Despite evidence of specific Np expression in human hippocampi and changed Np immunoreactivity in AD vs controls, it was not possible to determine a direct causal association of Np immunoreactivity and the estimated quantitative distribution of the neuropathological hallmarks of AD. This observation as well as a lack of significant correlation of quantified senile plaques or neurofibrillary tangles with age and duration of the disease, could be also explained by results of previous studies that pointed out great interindividual differences in neuropathological findings in ageing and AD,^19^ and difficulties in establishing a correlation between cell loss, NFT, and SP quantity.^18^, ^24^ However, observed relationship of hippocampal Np immunoreactivity in AD with the number of amyloid plaques and NFT in the present study seems promising for further investigation in a larger sample.

Interestingly, besides confirmed and previously described localization of Np expression on neuronal membranes, we observed intracellular Np immunoreactivity in subicular pyramidal neurons in AD. Although it is not possible to unequivocally explain this finding without further study, we may speculate that processing and/or trafficking of CAM neuroplastin is disturbed in vulnerable subicular areas affected by AD‐related neurodegeneration.^18^, ^19^

In conclusion, our preliminary results strongly indicate that altered hippocampal expression of cell‐adhesion glycoprotein neuroplastin in Alzheimer's disease is most probably related to a tissue plasticity response in neurodegeneration. Neuroplastin's role in the molecular mechanisms entailing neurodegenerative diseases is yet to be elucidated and requires further studies.

## CONFLICT OF INTEREST

The authors declare no conflict of interest.
