# Supplementary material for: Hippocampal expression of cell‐adhesion glycoprotein neuroplastin is altered in Alzheimer's disease
Source: J Cell Mol Med. 2018 Nov 28;23(2):1602–7. doi: 10.1111/jcmm.13998 (PMC6349345; doi:10.1111/jcmm.13998)
Supplement: Supplementary file 1 [file JCMM-23-1602-s001.docx]

SUPPORTING INFORMATION

Table S1. Individual data on subjects

| **C** | **Age (years)** | | **Gender** | **Cause of death** |
| --- | --- | --- | --- | --- |
| 1 | 62 | | F | Car accident |
| 2 | 68 | | M | Car accident |
| 3 | 75 | | M | Car accident |
| 4 | 71 | | F | Myocardial infarction |
| 5 | 81 | | F | Myocardial infarction |
| 6 | 84 | | F | Pulmonary embolism |
| **AD** | **Age (years)** | **Duration of disease (years)** | **Gender** | **Cause of death** |
| 1 | 73 | 4 | F | Bronchopneumonia |
| 2 | 73 | 7 | M | Bronchopneumonia |
| 3 | 77 | 3.5 | M | Bronchopneumonia |
| 4 | 80 | 5 | F | Bronchopneumonia |
| 5 | 80 | 6 | F | Myocardial infarction |
| 6 | 84 | 3.5 | F | Cardiovascular failure |

Brain sections were obtained from Huddinge Brain Bank, Karolinska Institutet, Stockholm, Sweden, with ethical and legislative permissions (The National Board of Health and Welfare Sweden, 5254-736/87; Regional Research Ethical Committee, Stockholm 024/01).

C - control subjects; AD - Alzheimer's disease

**Table S2.** Quantification of neuroplastin immunoreactivity in hippocampi derived from subjects with neuropathologically confirmed Alzheimer's disease and age-/gender-matched controls.

| **Hippocampal field** | **Layer** | **C** | **AD** |
| --- | --- | --- | --- |
| **Fascia dentata** | I stratum moleculare | ++ | ++/+++ |
|  | II stratum granulosum | ++ | ++ |
|  | III stratum plexiforme | + | ++ |
| **CA2/CA3** | Ia stratum moleculare | 0/(+) | 0/+ |
|  | Ib stratum lacunosum | + | +/(+) |
|  | Ic stratum radiatum | + | ++ |
|  | II stratum pyramidale | +/++ | ++ |
|  | III stratum oriens | 0/(+) | 0/+ |
| **CA1** | Ia stratum moleculare | 0/(+) | 0/+ |
|  | Ib stratum lacunosum | + | +/++ |
|  | Ic stratum radiatum | +/++ | ++ |
|  | II stratum pyramidale | ++ | ++/+++ |
|  | III stratum oriens | 0/(+) | 0/+ |
| **Subiculum (includes prosubiculum subiculum, presubiculum and parasubiculum)** | I stratum plexiforme | 0 | 0 |
|  | II stratum pyramidale parvocellulare | + | ++ |
|  | III stratum pyramidale magnocellulare | + | + |
|  | IV stratum pyramidale profundum | 0/+ | + |
|  | V stratum polymorfe | 0/+ | 0/+ |

Neuroplastin immunoreactive signal was semiquantitatively estimated in hippocampal sublayers by three independent researchers, *i. e.* by inspection of at least three tissue sections derived from control subjects (C) and Alzheimer’s disease (AD) showing satisfactory quality for light-microscopy analysis. Signal intensities: 0 - no immunoreactivity; + low immunoreactivity; ++ moderate immunoreactivity; +++ strong immunoreactivity.
